# Three-dimensional functional gradients direct stem curling in the resurrection plant *Selaginella lepidophylla*

**DOI:** 10.1098/rsif.2019.0454

**Published:** 2019-10-30

**Authors:** Véronique Brulé, Ahmad Rafsanjani, Meisam Asgari, Tamara L. Western, Damiano Pasini

**Affiliations:** 1 Department of Biology, McGill University, 1205 Avenue Docteur Penfield, Montréal, QC, Canada H3A 1B1; 2 Department of Mechanical Engineering, McGill University, 817 Sherbrooke Street West, Montréal, QC, Canada H3A 0C3; 3 Department of Materials, ETH Zürich, 8093 Zürich, Switzerland; 4 Theoretical and Applied Mechanics Program, Northwestern University, Evanston, IL 60208, USA

**Keywords:** biomimetics, resurrection plants, actuation, morphing, functional gradients, functionally graded materials

## Abstract

Upon hydration and dehydration, the vegetative tissue of *Selaginella lepidophylla* can reversibly swell and shrink to generate complex morphological transformations. Here, we investigate how structural and compositional properties at tissue and cell wall levels in *S. lepidophylla* lead to different stem curling profiles between inner and outer stems. Our results show that directional bending in both stem types is associated with cross-sectional gradients of tissue density, cell orientation and secondary cell wall composition between adaxial and abaxial stem sides. In inner stems, longitudinal gradients of cell wall thickness and composition affect tip-to-base tissue swelling and shrinking, allowing for more complex curling as compared to outer stems. Together, these features yield three-dimensional functional gradients that allow the plant to reproducibly deform in predetermined patterns that vary depending on the stem type. This study is the first to demonstrate functional gradients at different hierarchical levels combining to operate in a three-dimensional context.

## 1. Introduction

Functional gradients are the foundation of many biological processes, ranging from the migration of unicellular organisms through to influencing mechanical responses in complex, multicellular organisms [1,2]. Functionally graded materials (FGMs) result from gradual or stepwise changes in structure and/or composition that are tailored to alter their physical properties over a given volume [3]. In organisms, variations in material properties lead to programmed responses to internal and external stimuli. In biomimetics, FGMs are sought after because they often display enhanced function or improved functional longevity over non-FGMs [4,5].

Plant tissues can be considered as FGMs whose structure and composition are tailored to specific functions, including seed dispersal, predation and predator evasion [6,7]. One of the most fascinating examples of FGM-based function in plants is actuation: the process of autonomous deformation that is triggered by an external stimulus [6,8]. Biological actuators, such as the pinecone, the seeds of the ice plant and the wheat awn undergo a set of well-defined and reproducible shape transformations as part of their physiological response to changes in hydration status [6,8–12]. Deformation in these species arises from differential swelling/shrinking of juxtaposed tissues with distinctive material properties [12–15]. Similarly, *Selaginella lepidophylla*, a resurrection spikemoss that can tolerate extreme drought conditions, also deforms through swelling and shrinking of its vegetative tissue in response to changes in relative water content [16–20]. However, unlike the plants listed above, in which deformation occurs in dead tissue, in *S. lepidophylla*, deformation is observed in both dead and living vegetative tissue and serves to limit the amount of photo, thermal and water-deficit stresses to which the plant is exposed during periods of drought [17–19]. Thus, as a biological actuator, *S*. *lepidophylla* provides a unique opportunity to explore and compare properties giving rise to the deformation of living and dead tissues within the same system.

**Figure 1.**
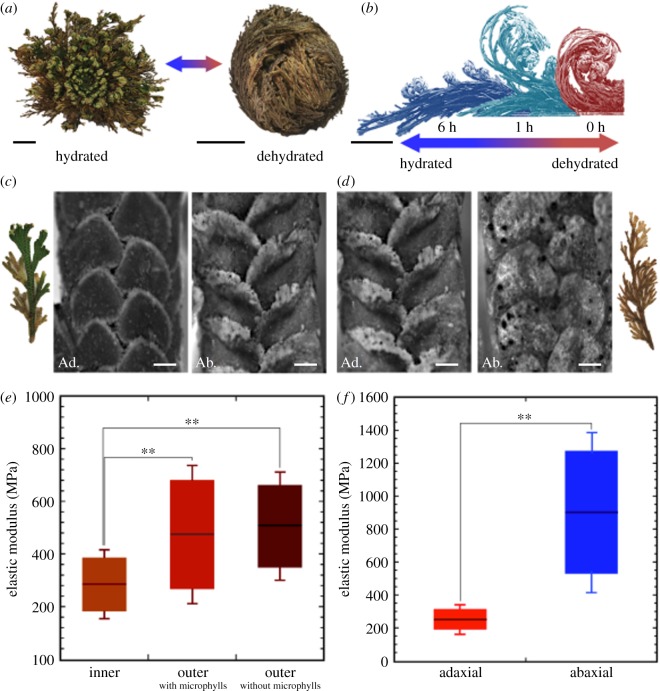
Conformational changes in *S. lepidophylla*, and organ and tissue stiffness. (*a*) *S. lepidophylla* plant in a hydrated conformation with opened, spirally arranged stems, and a dehydrated conformation showing outer stems curled and precisely packed over inner stems. Scale bars: 2 cm. (*b*) Time-lapse stills (0, 1 and 6 h after the onset of rehydration) showing uncurling of inner and outer stem types in response to a change in hydration state. Scale bar: 2 cm. (*c*) Inner stem (average length range = 3–6 cm), showing adaxial (Ad.) and abaxial (Ab.) stem sides including microphylls. Scale bars: 200 μm. (*d*) Outer stem (average length range = 6–12 cm), showing adaxial (Ad.) and abaxial (Ab.) stem sides including microphylls. Scale bars: 200 μm. (*e*) Boxplot comparing average stiffness with s.e. among inner and outer stem types (further information included in electronic supplementary material, table S2). (*f*) Boxplot comparing average stiffness with standard error between adaxial and abaxial cortical tissue of inner stems (further information included in electronic supplementary material, table S3). (Online version in colour.)

*Selaginella lepidophylla* is composed of hundreds of stems connected together by an extensive root system. These stems are arranged in a spiral phyllotaxy with developing (inner, living) stems at the centre of the plant and sequentially more mature (outer, dying-to-dead) stems spiralling outward from the centre. When hydrated *S*. *lepidophylla* stems are completely uncurled and the plant appears as a flattened rosette. Upon dehydration, stems curl and the whole plant deforms into a spherical shape, with outer stems curling over inner stems (figure 1*a,b*; electronic supplementary material, movie S1) [21,22]. Inner and outer *S. lepidophylla* stems curl to different degrees that, in combination with a spiral phyllotaxy, allow for tight and precise stem packing during desiccation-induced deformation. Preliminary investigation has suggested that asymmetric cell density and lignin distribution might contribute to the distinct degrees of curling and mechanical responses exhibited by inner and outer stem types [22]. However, other unexplored factors may control water-induced shape transformations in *S. lepidophylla*.

In this paper, we adopt a broad experimental approach to fully elucidate the role of functional gradients leading to unique deformation patterns in *S. lepidophylla*. In particular, we aim to address two related questions: (1) what properties lead to directional stem deformation and (2) how do these properties contribute to the different degrees of curling observed in inner and outer *S. lepidophylla* stem types? We take advantage of an array of techniques to explore how morphology (micro-computed X-ray tomography and transmission electron microscopy (TEM)), composition (histochemistry and immunofluorescence microscopy) and mechanical properties (microtensile testing and nano-indentation) at the tissue and cell wall levels lead to deformation in *S. lepidophylla*. We show that a combination of morphological and compositional properties gives rise to three-dimensional functional gradients that drive water-responsive shape transformation in *S. lepidophylla*, and that the variation in curling of inner and outer stem types results from specific combinations of functional gradients.

## 2. Material and methods

### 2.1. Plant materials

*Selaginella lepidophylla* were acquired and maintained as described in [22].

### 2.2. Time-lapse video capture

Time-lapse video capture for electronic supplementary material, movies S1 and S2, and figure 1*b* was adapted from the procedure described in [22]. Wedge-shaped portions of representative *S*. *lepidophylla* plants were isolated and allowed to either air dry to a fully dehydrated state or to rehydrate over the course of 6 h. Changes in stem deformation were recorded over the course of approximately 6 h (electronic supplementary material, movie S1). Individual inner and outer stems were isolated and subjected to repeated wetting and drying to demonstrate the reversibility of deformation over multiple cycles of rehydration and dehydration (electronic supplementary material, movie S2).

### 2.3. Stem and tissue tensile testing

Twenty *S. lepidophylla* plants were rehydrated to 100% relative water content. For whole stem tests, 75 stems were isolated randomly from these 20 plants: 25 inner stems, 25 outer stems with microphylls and 25 outer stems without microphylls. For adaxial/abaxial region tests, 50 inner stems were isolated randomly and cut lengthwise (25 adaxial/abaxial, 25 left/right stem sides) and the vascular bundle (VB) removed. Stems were secured between clamps of an ADMET MicroEP machine with the base of the stem always clamped at the load cell end. A 10 lb load cell was used for testing. Stems were tested in a hydrated state for sample manipulation, as stems become fragile with water loss and tend to break when clamped into the tensile testing apparatus. Stems were pulled at a rate of (10 mm min^−1^) until failure. Stem thickness, width and length were measured prior to testing (electronic supplementary material, table S1). Load and displacement were recorded using MTESTQuattro software.

### 2.4. Light microscopy

Five, fully hydrated *S. lepidophylla* stems were isolated from three different plants and embedded in polyethylene glycol (PEG) using the protocol from [23]. Embedded samples were then sectioned (10 µm thickness) using a Leica RM2245 semi-automated rotary microtome. Solidified PEG was then removed using washes of ddH_2_O. One set of samples was mounted, unstained, and the set was stained with Toluidine Blue O following the protocol in [24]. Samples were mounted in ddH_2_O and slides were sealed with nail polish to prevent water from evaporating. Samples were examined using a Leica DM6000B epifluorescence microscope with the brightfield setting (10× and 40×), and images were acquired using a Qimaging Retiga CCD camera operated through Openlab.

### 2.5. Transmission electron microscopy

Ten inner and 10 outer stems were isolated from five hydrated plants. Sections of 2 mm in length corresponding to apical, middle and basal regions of the stem were cut from the 10 samples. Five replicates from each stem region were immediately placed in a fixing solution, and another five replicates were allowed to air-dry overnight to a completely dried state. Stem sections were fixed and embedded in Spurr's Resin following the protocol outlined in [22]. Sample blocks were then thin sectioned with a Leica EM UC6 ultramicrotome using an Ultra 45 DiaTOME knife (clearance angle of 6°, speed of sectioning 0.8 mm s^−1^, feed 70 nm). Sections were transferred to copper, formvar-coated grids (200 mesh or 0.4 × 2 mm slotted) and allowed to air dry. Imaging was performed at the Facility for Electron Microscopy Research at McGill University using an FEI Tecnai 12 BioTWin 120 kV TEM, equipped with an AMT XR80C CCD camera system.

### 2.6. Micro-computed X-ray tomography

The three-dimensional anatomy and microstructural features of freshly cut inner and outer stems of *S. lepidophylla* were acquired by performing a series of synchrotron radiation-based phase-contrast X-ray tomographic microscopy experiments, a non-destructive and high-spatial-resolution imaging technique, at the TOMCAT beamline of the Swiss Light Source at the Paul Scherrer Institute (Villigen, Switzerland) [25]. The TOMCAT beamline exploits a 2.9 T magnetic dipole with a critical energy of 11.1 keV. A double crystal multilayer monochromator is used to select X-rays with a central X-ray photon energy of 15 keV. The X-rays, after interacting with the sample, are converted into visible light using a LuAG : Ce 20 mm scintillator. This light passes through a 10× optical microscope, which is reflected in a mirror and finally captured on a 2560 × 2160 pixels sCMOS camera (PCO.Edge 5.5). As the woody tissue of the stems of *S. lepidophylla* is composed of elements with small atomic numbers, i.e. carbon, oxygen and hydrogen, it has a low X-ray attenuation coefficient. Therefore, the phase-contrast tomography method was used in which the samples were subjected to a very low dosage of X-ray beam energy. Here, the source of phase contrast was the difference in the X-ray index of refraction caused by the density gradient at the material–air interface. During each tomography run, 1701 projections were collected over 180° at an exposure time of 120 ms per image, resulting in a total scanning time of approximately four minutes. The simultaneous phase and amplitude retrieval algorithm was used for tomographic reconstruction [26]. After reconstruction, a tomographic dataset consists of 2160 slices stacked at one-pixel interval along the axial direction and each cross-sectional slice has 2560 × 2560 pixels. With an isotropic voxel size of 0.65 µm^3^, the approximate field of view is about 1664 × 1664 × 1404 µm^3^.

### 2.7. Atomic force microscopy

A JPK atomic force microscope (JPK Nano-wizard@3 Bio Science, Berlin, Germany) was used for imaging and force spectroscopy. To prepare the samples for atomic force microscopy (AFM), five *S. lepidophylla* stems were cut transversely by a double-edged blade. Sections (1 mm thickness) were air-dried to a dehydrated state to remove the influence of turgor on cell wall indentation measurements. Cut, dried sections were placed on double-sided clear tape on a microscope slide. Cortical stem tissue in adaxial and abaxial regions was located to perform force measurements. The AFM measurements were performed in an ambient environment of 20–25°C and less than 30% RH (note: *S. lepidophylla* does not absorb moisture from the air; thus, sections remained in a dehydrated state for the duration of testing). Using the QI imaging mode of the JPK AFM, a force map was created within an area of 30 µm^2^ on the sample. Reduced scan areas were then selected to obtain the structural details of the cell walls in which 128 × 128 indentation points were tested in areas of 1–10 µm^2^. The force maps averaged 3 µm^2^ in size. For consistency, only the areas (less than 5–9 per cell) located on selected parts were indented. For contact mode imaging, non-conductive silicon nitride cantilevers with integrated spherical tips of radius 20 nm (MLCT Micro-cantilever, Bruker, Mannheim, Germany), and super-sharp standard Force Modulation Mode Reflex Coating cantilevers with diamond-like carbon nano-tip of radius 2–3 nm (Nanotools USA LLC, Henderson, NV) were used. For indentation measurements, non-contact high-resonance cantilevers (Nanotools USA LLC, Henderson, NV) with a nominal spring constant of 40 N m^−1^ and integrated spherical tip of radius 100 nm (±10%) were applied. The deflection sensitivity of the piezo module was obtained by probing the surface of a glass substrate.

To calibrate the stiffness of the cantilever, the first free resonance peak of the cantilever was fitted to the equation of a simple harmonic oscillator to obtain the power spectral density of thermal noise fluctuation in the air [27,28]. The indentation was repeated at the same location for consistency as well as to ensure that the sample was not permanently deformed [29,30]. The indentation depth depends on the applied load, as well as the stiffness of the tip and that of the sample. Here, an indentation frequency of 250 Hz was used. The elastic modulus *E* of a sample was estimated from the retracting force–indentation depth curve through Hertzian contact mechanics, where *E* = 3*F* (1 − *ν*^2^)/4 *Rδ*^3^ is the relation between the elastic modulus *E* and the applied indenting load *F* with *ν* being the Poisson's ratio of the sample, *R* the radius of the AFM probe and *δ* the indentation depth [31]. A number of assumptions were considered. The deformation of the sample relative to its thickness and also relative to the radius of the probe was assumed to be very small. Any strain below the elastic limit was also assumed infinitesimal, a condition satisfied with the use of an indentation depth below 50 nm that rules out the influence of the glass substrate as well as any nonlinear and inelastic behaviour of the tissue at higher strains. The Poisson's ratio *ν* was selected to be 0.5. AFM data analysis was performed with the native JPK data processing software. Statistical significance was determined by a paired Student's *t*-test, when applicable. Differences were considered significant at *p* < 0.05.

### 2.8. Immunohistochemical staining

Ten inner and 10 outer stems were randomly isolated from 5 hydrated *S. lepidophylla* plants, and 2 mm sections were cut from the apical, middle and basal regions of each stem. Stems were fixed and embedded in London-Resin (LR) White following the protocol from [32]. LR White-embedded samples were then semi-thin sectioned (500 nm, feed of 25 mm s^−1^) using a Leica EM UC6 ultramicrotome. Sections were placed onto Teflon-coated slides (EMS #63424-06) and allowed to air-dry on a covered slide-heater at 40°C overnight. Samples were immunostained following a protocol adapted from [32]. Antibodies used are described below in the section ‘Primary and secondary antibodies'.

Samples were incubated at room temperature in a blocking solution (5% (w/v) normal goat serum (NGS) in 1× Tris-buffered saline/0.2% Tween (v/v) (TBST)) in a homemade humidity box for 40 min. Blocking solution was washed off using 1× TBST. Slides were then incubated with primary antibodies at 1 : 10 dilution (v/v) in 1% (w/v) NGS blocking solution in the humidity box for 1 h. Slides were washed 2 × 20 min in 1× TBST. Secondary antibodies were diluted at 1 : 100 (v/v) in 1% (w/v) NGS blocking solution in the dark for 45 min. Slides were washed 2 × 20 min in the dark and mounted in 90% (v/v) glycerol. Control slides were used to test the specificity of the secondary antibodies and to test for autofluorescence. Blocked slides that were not incubated with primary or secondary antibodies were imaged, as well as slides blocked and incubated with only secondary antibody.

For examination of lignin, samples were prepared (with basic fuchsin) as described in [22].

Samples were examined using a Leica DM6000B epifluorescence microscope, and images were acquired using a Qimaging Retiga CCD camera operated through Openlab. The following channels were used: YFP (immunofluorescence imaging of LM10 and LM11), GFP and TX2 (for detection of basic fuchsin [33,34]).

### 2.9. Primary and secondary antibodies

Previously published LM10 (Rat IgG2c) and LM11 (Rat IgM) antibodies were obtained from Plant Probes, UK [35]. These anti-rat monoclonal antibodies were generated against (1→4)-d-xylans. LM10 binds to unsubstituted or low-substituted xylan backbone chains, while LM11 is able to additionally bind to wheat arabinoxylan. Alexa-fluor 488 secondary antibody was obtained from Invitrogen (goat anti-Rat IgG (H + L) polyclonal, CAT# A-11006).

### 2.10. Statistical analysis

All statistical analyses were performed using RStudio for Mac (v. 1.1.383 © 2009–2017 RStudio, Inc.).

## 3. Results

### 3.1. Gradients of elastic modulus in *S. lepidophylla* inner and outer stems

The degree of curling of individual *S. lepidophylla* stems varies with their location in the plant's spiral phyllotaxy (figure 1*a,b*) [22]. Time-lapse observation of dehydrating plants reveals that inner stems curl slowly into tight spirals, whereas outer stems rapidly curl into an arc shape (electronic supplementary material, movies S1 and S2) [22]. Given these specific curling profiles, we expect the mechanical properties involved in deformation to also vary between inner and outer stem types. We performed uniaxial tensile tests on hydrated inner (figure 1*c*) and outer stems (figure 1*d*), with outer stems being divided between those that have lost their leaflets (microphylls) and those that retained them, as leaflets can influence surface water movement along the stem. Mechanical properties were calculated from stress–strain plots obtained during testing (electronic supplementary material, table S2). Of particular interest is the elastic modulus (*E*), which measures the ability of a material or object to resist deformation under an applied force [36,37]. High *E* values denote stiff materials that resist deformation, while low values are common for flexible materials that readily deform under load. Mechanical testing of *S. lepidophylla* stems revealed that, in a fully hydrated state, outer stems, with and without microphylls, are significantly stiffer than inner stems (figure 1*e*; electronic supplementary material, table S2).

During dehydration, *S*. *lepidophylla* stems curl toward their adaxial (upper) side, as opposed to their abaxial (lower) side (electronic supplementary material, movies S1 and S2). As this movement can be represented with a simple bilayer composite model [22], we hypothesized that adaxial and abaxial stem sides differ in their relative stiffness. Hydrated, inner stems were cut lengthwise, and adaxial and abaxial sides were subjected to uniaxial tensile testing (electronic supplementary material, table S3). The abaxial region was significantly stiffer than the adaxial region (figure 1*f*). As a control, inner stems were cut lengthwise into the left and right sides and tested. No significant difference in stiffness was observed (electronic supplementary material, table S3). Due to their brittleness, outer stems could not be cut and similarly tested.

The results above show that, at the organ level, the degree of stem curling observed in inner and outer stems appears to be associated with differences in stiffness. In addition, a stiffness gradient between adaxial and abaxial stem sides seems to contribute to directional bending in inner stems, whereby the stem curls toward the less stiff (adaxial) side. Given the possible contribution of stiffness gradients to the direction and extent of stem curling, we decided to investigate the underlying features responsible for differences in stiffness between inner and outer stems and also between adaxial and abaxial stem sides.

### 3.2. Transverse morphological gradients exist across adaxial and abaxial stem regions

**Figure 2.**
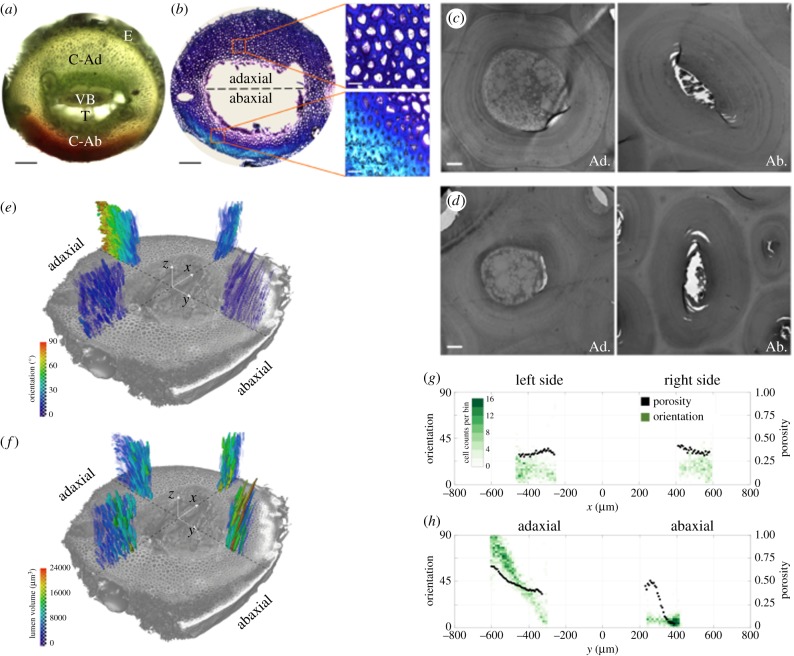
Morphology and tissue structure in *S. lepidophylla*. All images are from the apical region of inner stems with the exception of (*d*) which shows inner basal cells. (*a*) Unstained cross-section showing tissue morphology. E, epidermis; C, cortex (Ad, adaxial; Ab, abaxial); VB, vascular bundle; T, trabeculae. Scale bar: 200 µm. (*b*) Toluidine Blue O-stained cross-section showing changes in tissue thickness and cell density between adaxial and abaxial cortex. Scale bars: 200 µm, and 50 µm (insets). (*c,d*) Transmission electron microscopy images of (*c*) apical cortex and (*d*) basal cortex cell shape near the centre of the plant where cell orientation is parallel to the longitudinal axis. Scale bars: 2 µm. (*e,f*) Three-dimensional micro-computed X-ray tomography reconstructions showing a colour map (*e*) of cortex cell orientation relative to the longitudinal axis, and (*f*) lumen volume between adaxial and abaxial, as well as left and right stem sides. (*g,h*) Quantification of normalized cell orientation and porosity of cortical tissue are shown for left and right (*g*) and adaxial and abaxial (*h*) stem sides. On the *x*-axis, zero represents the centre of the stem and the relative distance (in μm) that individual cells are from the stem centre. (Online version in colour.)

*Selaginella lepidophylla* stems are composed of four main tissue types (figure 2*a*). Microphylls are attached to a thin epidermal layer covering a thick cortical tissue layer. The cortex is shaped like a hollow cylinder surrounding an amphicribral VB that runs through an airspace in the centre of the stem. The VB is connected to the cortex by large, thin-walled trabeculae cells. As the bulk of the stem is made up of cortex, we focused on comparing the morphology of this tissue between inner and outer stems and between adaxial and abaxial stem sides.

Light microscopy revealed two differences in adaxial and abaxial cortical tissue across all observed regions (apical, middle and basal) of inner and outer stems. First, the adaxial cortex is thicker than the abaxial cortex (figure 2*b*) [22]. This was also observed when recording stem dimensions of adaxial and abaxial regions for mechanical testing (electronic supplementary material, table S1). Second, the abaxial cortex appears denser (more cells per square area) when compared to the adaxial cortex (figure 2*b* insets).

TEM was performed to further investigate the difference between adaxial and abaxial cortex at the cellular level. Both cell size and shape were examined. Average cross-sectional cell area, cell wall area and lumen area were calculated for apical, middle and basal inner stem regions. Adaxial cortical cells had a significantly larger total cell area and lumen area across all stem regions when compared to abaxial cortical cells (table 1, figure 2*c,d*). By contrast, cell shape did not change significantly between adaxial and abaxial cortical cells, or across cells in apical, middle and basal stem regions (figure 2*c,d*).

**Table 1.** Adaxial and abaxial cortical tissue cell dimensions (mean ± s.e.) of inner stems. TEM images were used to quantify (in μm) total cell area, as well as cell wall/lumen area and cell wall thickness. Differences between adaxial and abaxial cell dimensions were tested with paired Student's *t*-tests with a cut-off of *p* = 0.05. One hundred cells were measured for each stem and tissue region. Significant results (i.e. *p* < 0.05) are marked by +.

	apical		middle		basal	
cell dimensions	adaxial	abaxial	adaxial	abaxial	adaxial	abaxial
total cell area	290.60 ± 16.19^+^	203.00 ± 10.81^+^	274.86 ± 12.02^+^	230.32 ± 11.49^+^	247.33 ± 9.36^+^	215.02 ± 9.52^+^
cell wall area	201.84 ± 10.15	182.63 ± 9.57	245.45 ± 10.43	217.91 ± 11.02	226.24 ± 8.50^+^	202.52 ± 9.09^+^
lumen area	88.76 ± 6.89^+^	20.38 ± 2.19^+^	29.41 ± 2.50^+^	12.41 ± 0.80^+^	21.09 ± 1.47^+^	12.50 ± 0.73^+^
cell wall thickness	3.95 ± 0.1^+^	5.19 ± 0.15^+^	5.68 ± 0.13	6.08 ± 0.12	5.88 ± 0.12	5.81 ± 0.14

Three-dimensional stem tissue morphology was assessed using high-resolution synchrotron radiation phase-contrast X-ray tomographic microscopy, and three-dimensional image analysis. Four radial sections were analysed across apical and basal regions of inner stems: adaxial, abaxial, left and right (figure 2*e,f*). In each region, which stretches from the periphery toward the centre of the cortex, cell orientation with respect to the long axis of the stem was determined, as well as the tissue porosity, which is defined by the ratio of the lumen volume to the total volume of the region of interest. While there were minor variations between the left and right sides of the stem cortex in terms of three-dimensional structure (figure 2*e–g*), significant differences in the microstructure were seen between adaxial and abaxial sections (figure 2*e–h*). First, abaxial cortical cells are aligned parallel with the longitudinal stem axis, whereas adaxial cortical cells change orientation from being nearly aligned along the stem axis in the inner side of the cortex to being perpendicular to this axis at the periphery (figure 2*e,h*). Second, there are alterations in tissue porosity. The left and right regions exhibit a symmetric and almost constant porosity profile which slightly increases from the periphery to the inner side of the cortex (figure 2*g*). By contrast, the porosity of adaxial and abaxial tissues and its variation within each of these tissues are substantial. Abaxial tissue is almost fully solid at the periphery and becomes more porous toward the inner side of the cortex. In general, adaxial tissue is wider and more porous than abaxial tissue and its porosity increases from the inner side of the cortex towards the periphery (figure 2*e,h*). This is also consistent with the lower cell density observed in adaxial tissue with light microscopy (figure 2*b*).

Taken together, structural differences exist between adaxial and abaxial cortex, as observed from tip to base in both inner and outer stems, though only images from the apical region of inner stems are shown here as representative examples. Based on these results, we decided to examine cortical cell walls to identify whether or not there were also differences in adaxial and abaxial stem sides visible at the cell wall level.

### 3.3. Transverse gradients of secondary cell wall properties exist in both inner and outer stems, but longitudinal gradients only exist in inner stems

**Figure 3.**
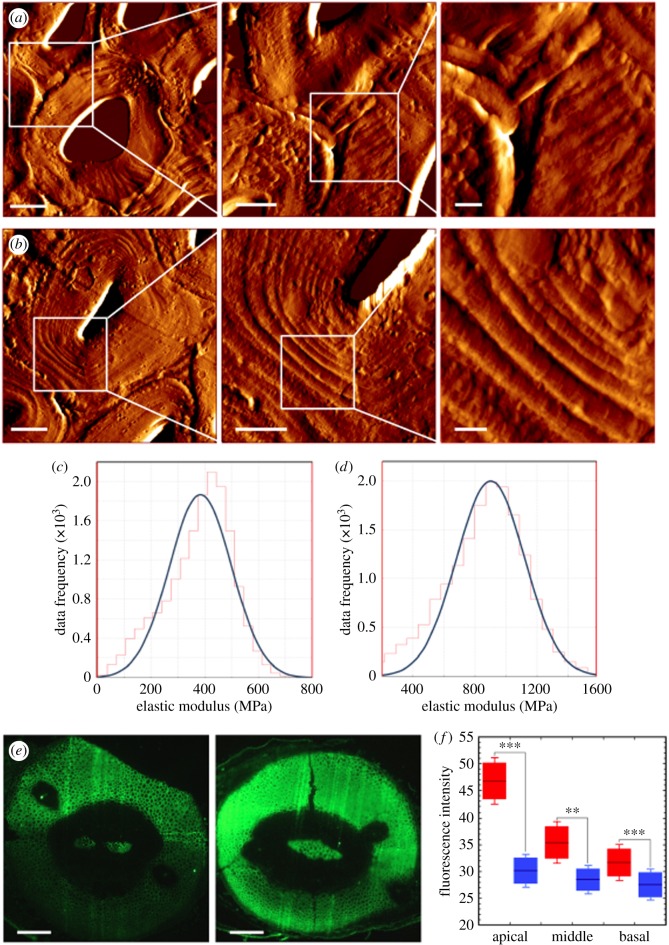
Cell wall properties in *S. lepidophylla*. (*a,b*) Atomic force microscopy images of apical, inner stem adaxial (*a*) and abaxial (*b*) cells. Scale bars from left to right represent 5 µm, 2 µm and 500 nm. (*c,d*) Elastic modulus distribution showing average stiffness values based on representative populations of 5–10 cells for apical adaxial (*c*) and abaxial (*d*) cell types. (*e*) Immunofluorescence results for inner stem apical (left) and basal (right) regions showing LM11 binding pattern. Scale bar: 200 µm. (*f*) Boxplot showing average fluorescence intensity (LM11) and standard error between adaxial (left box of each pair) and abaxial (right box of each pair) tissue regions for apical, middle and basal stem cross-sections. Asterisks indicate significant (**p < 0.05, ***p < 0.01) differences in fluorescence intensity between adaxial and abaxial stem sides. (Online version in colour.)

Cell wall thickness is a known factor that affects plant stiffness [37,38]. Quantification of cell wall thickness using TEM revealed that, between adaxial and abaxial cortex cells along the length of the stem, abaxial cell walls are significantly thicker in apical cell walls, but this difference between stem sides is lost in the middle and basal portions of inner stems (figure 2*c,d*, table 1). This pattern of cell wall thickness was also observed in topological scans of hand-cut, dried inner sections imaged using AFM (figure 3*a,b*). Likewise, both AFM and TEM showed similar results with regard to cell shape and lumen size (compare figure 2*c,d* with figure 3*a,b*). In addition to examining cell morphology, nano-indentation was performed to explore cell wall stiffness. As seen at the gross level for longitudinally cut whole stems, apical adaxial cell walls are significantly less stiff than apical abaxial cell walls (340 and 870 MPa respectively, *p* < 0.05) (figure 3*c,d*).

In addition to wall thickness, cell wall stiffness is also affected by the types, quantities, localization and interactions between various wall polymers. These include not only cellulose but also the matrix polysaccharides (pectins and hemicelluloses) and the polyphenolic lignin [38,39,40]. The presence and distribution of these polymers were examined using a combination of fluorescence microscopy and immunohistochemistry. Lignin has previously been shown to be present in *S. lepidophylla* stems, with differences not only between adaxial and abaxial cortex but also across apical, middle and basal inner stem regions [22,41]. At the stem apex, basic fuchsin staining detected lignin in the abaxial cortex near the stem periphery; in the stem middle, lignin was observed throughout the abaxial cortex; and at the stem base, both adaxial and abaxial cortex were uniformly lignified (electronic supplementary material, figure S1). Lignification in outer stems was consistent from stem tip to base and was distributed throughout both adaxial and abaxial cortical tissue [22].

To evaluate the presence, location and amount of pectic and hemicellulosic polysaccharides, inner *S. lepidophylla* stems were subjected to immunostaining with a battery of pectin and hemicellulose-detecting antibodies. Of the 11 pectin and 9 hemicellulose antibodies used, 10 bound to *S. lepidophylla* sections. In most cases, the antibodies either bound only to the VB and/or showed uniform binding across adaxial/abaxial cortex and/or along the stem (electronic supplementary material, table S4). However, two antibodies detecting secondary cell wall hemicellulose, LM10 and LM11 [35,42], differed in epitope binding both between adaxial and abaxial cortex, and along the apical–basal stem axis. LM10 (unsubstituted/low substituted xylan) and LM11 (unsubstituted/low/highly substituted xylan) have overlapping binding patterns in *S. lepidophylla* stems (figure 3; electronic supplementary material, figure S2). In apical regions, both antibodies bind strongly to adaxial cells and only weakly to abaxial cells. While antibody binding to adaxial cells decreases to a certain extent in middle and basal stem cross-sections, it still remains significantly higher than that seen in the abaxial side (figure 3*e,f*; electronic supplementary material, figures S2 and S3). LM10 and LM11 antibodies bind in a similar pattern to each other in the adaxial and abaxial cortex of both inner and outer stems. However, outer stems do not show decreasing tip–base antibody binding. Rather, binding remains consistent among apical, middle and basal cross-sections and is similar to that seen in basal sections from the inner stem (electronic supplementary material, figure S3).

## 4. Discussion

**Figure 4.**
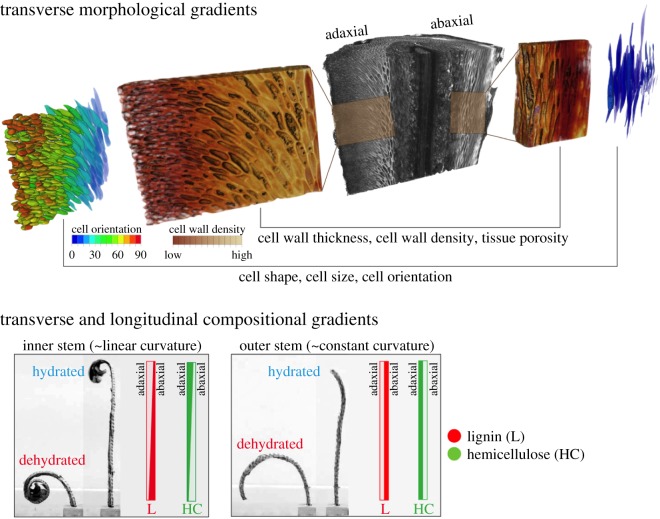
Summary diagram of the morphological and compositional gradients that contribute to directional bending and the degree of stem curling in *S. lepidophylla*. Inner stems curl tightly into a spiral shape upon drying, whereas outer stems curl into an arc shape upon drying. Directional bending relies primarily upon structural gradients (morphology), including cell orientation, cell size and tissue porosity that lead to changes in tissue density between adaxial and abaxial cortex. The difference in the degree of stem curling between inner and outer stem types is most likely driven by tip-to-base compositional gradients that include variation in lignification (L) and hemicellulose distribution (HC) in the cell wall. Outer stems do not show tip-to-base gradients whereas inner stems do, and do not curl as tightly as inner stems. (Online version in colour.)

Previously, we examined the deformation patterns (rates, direction and forces involved) of *S. lepidophylla* plants as a response to water intake and loss [22]. Here we show that directional bending, as well as the variation in the degree of curling between inner and outer rosette stems, is a result of a complex three-dimensional system of transverse and longitudinal functional gradients arising from multiple structural and compositional gradients observed at tissue and cell wall levels. We present data suggesting that directional curling results primarily from structural gradients across the stem cross-section, from adaxial to abaxial sides. In the case of inner stems that show a more complex curling pattern, gradients also exist along the length of the stem. Figure 4 provides a summary of the features leading to directional stem bending and the degree of bending in *S. lepidophylla*. To the best of our knowledge, this is the first study to demonstrate functional gradients at different hierarchical levels combining to operate in multiple three-dimensional contexts within the same structure.

### 4.1. Directional deformation of *S. lepidophylla* stems is associated with transverse gradients of tissue morphology and cell wall properties

Mechanical testing of dissected adaxial and abaxial (but not left and right) sides of *S. lepidophylla* stems revealed that the adaxial portion of the stem is less stiff (figure 1*f*). This is consistent with the proposed bilayer model that supposes the juxtaposition of two layers with differential swelling (shrinking) in response to stem hydration (dehydration). The active layer generally drives tissue/organ movement, while the passive layer constrains and controls the direction of movement [6,9,12,43–45]. In *S. lepidophylla*, the less stiff adaxial tissue would comprise the active tissue whose greater swelling/shrinking in response to hydration status would push or pull the stiffer, passive abaxial tissue into a different conformation. What underlying properties contribute to this differential stiffness, and how do they influence directional bending of *S. lepidophylla* stems? Detailed analysis of the cortical tissue at several length scales and in both two and three dimensions suggests that there is a complex morphological and biochemical hierarchy involved.

Observed structural differences between adaxial and abaxial cortical tissue in *S. lepidophylla* can be divided into cell size and shape, secondary cell wall composition and cell angle with respect to the primary stem axis. While the abaxial cortical cells are elongated, thick-walled cylinders with relatively narrow lumens, adaxial cells tend to be shorter and wider (larger cross-sectional lumen area). This leads to higher tissue density in the abaxial cortex (more cells per square area), which can contribute to increased stiffness. Conversely, immunohistochemistry revealed that across stem types, adaxial cells have a greater proportion of hemicellulose xylans, which could contribute to increased swelling of the adaxial cortex (see below for more discussion of cell wall composition). In addition, the orientation of adaxial cells with respect to the primary stem axis varies significantly from the inner side of the cortex (parallel) toward the periphery (perpendicular), while abaxial cells lie parallel to the longitudinal axis. This establishes differential swelling/shrinking angles between the two sides of the stem, leading to bending. In terms of the direction of bending, given the larger lumen cross-sectional area and lower tissue density of the adaxial cortex, it is expected to shrink/swell on an angle and to a greater extent than the abaxial cortex in response to changes in water status. This would result in pulling toward the adaxial side with dehydration (shrinkage), and thus adaxial bending, and pushing toward the abaxial side with hydration (swelling), leading to straightening of the stem.

### 4.2. Lengthwise gradients of cell wall thickening, lignification and hemicellulose are associated with linear curling in inner *S. lepidophylla* stems

Structural, immuno- and histochemical analyses suggest that tissue morphology is identical between inner and outer stems and that the base of inner stems is equivalent to the whole length of the outer stem. What differs between stem types, and what gives rise to tighter curling of inner stems as compared to outer stems, are the characteristics of the cell walls along the length of the inner stems, namely, tip-to-base gradients in cell wall thickness and composition. In the apical region of inner stems, abaxial cortex cells have thick secondary cell walls, whereas adaxial cell walls are thinner. In the middle of the stem, this difference is lost (table 1). Compositionally, there is a gradient of lignification along the inner stems in which lignin is most strongly detected along the outer curve of the apical abaxial cortex, throughout the whole abaxial cortex mid-stem and across the entire (adaxial and abaxial) cortex at the stem base (electronic supplementary material figure S1). Conversely, antibody detection of hemicellulose xylans reveals a mirrored gradient (figure 3; electronic supplementary material figure S2). Xylans stain strongly in the adaxial portion of the cortex at the apical region and become less apparent lower in the stem (they are consistently detected at a low level in the abaxial cells). Considered from a developmental viewpoint, secondary cell wall differentiation could be seen as delayed in the adaxial cortex.

Both secondary cell wall thickness and composition affect cell and tissue stiffness [38,40]. In walls of equal composition, thicker walls act to strengthen and stiffen. The deposition of lignin plays a similar role. A polyphenolic polymer, lignin coats the polysaccharides and fills in the pores of cell walls, making them both stiffer and hydrophobic [38,46]. Thus, lignified tissue is less elastic and less able to swell/shrink in response to changes in hydration status. The significance of the gradient of xylan detection is less obvious. It is possible that it is present throughout all cortical cell walls in an equal amount, and the strong binding of the antibodies seen in the apical adaxial region of inner stems (and to a lesser extent in all abaxial regions) is a reflection of decreased levels of lignin in these regions—i.e. the presence of lignin masks the hemicellulose epitopes. Another possibility is that xylan acts as a plasticizer to promote tighter and reversible cell wall compaction during dehydration, as suggested for other resurrection plant species [47–49]. Xylan could be binding to cellulose microfibrils to replace hydrogen bonds during dehydration as cells lose water. This would prevent microfibrils from binding to each other, allowing for reversible cell wall compaction leading to more tissue shrinking and larger deformation at the organ level [48–51]. Higher abundance of xylan in the adaxial cortex corresponds with the role of the adaxial side as an active layer that pushes and pulls the abaxial, passive layer during stem deformation. A decreasing tip-to-base gradient of xylan abundance in adaxial cortex also fits with the observed pattern of tight curling at the stem tip and less curling at the stem base. This appears to be a reasonable function for xylan with respect to *S. lepidophylla* stem curling because outer stems show lower adaxial xylan abundance and consistent xylan staining from tip to base, and they are unable to curl to the same degree as inner stems. However, further testing to quantify xylan and lignin abundance and map their spatial distribution in adaxial and abaxial tissue is needed to more precisely determine the presence of xylan in *S. lepidophylla* cortical cell walls and its function in stem deformation.

The sum of these properties suggests that inner stems have a stiffness gradient from tip to base. Stems are less stiff at the tip where the adaxial cortex cell walls are thinner and lignification is only significant on the most abaxial edge of the cortex, and they become progressively stiffer moving toward the stem base where the adaxial cell walls thicken and become lignified. As well, higher accessibility of hydrophilic hemicellulose epitopes in the adaxial cortex suggests a greater response to water gain/loss in this tissue as compared to the abaxial cortex. Together, these features would allow for tighter curling at the tip and progressively less curling moving downward, resulting in a spiral shape with stem drying.

### 4.3. Comparison of functionally graded material-based deformation in *S. lepidophylla* to other established plant models

Functional gradients are observed in a number of water-driven actuating plant species, including—but not limited to—pinecones [12,52,53], wheat awns [13,14,43] and orchid tree seedpods [43,54]. The juxtaposition of active and passive tissue layers in these species gives rise to differential tissue swelling and shrinking, leading to movement such as bending. However, unlike these species with very distinct, one-dimensional bilayer gradients, in *S. lepidophylla*, there is an added degree of complexity in that the functional gradients are three-dimensional (transverse and longitudinal), spanning from stem tip to base and/or between stem sides (adaxial/abaxial). In this aspect, *S*. *lepidophylla* somewhat resembles bamboo; both show transverse and longitudinal gradients leading to specific mechanical behaviours to counteract environmental stresses imposed upon the plant. In bamboo, cellulose fibres confer mechanical support to the culm. Fibres are arranged in a radial pattern, with increasing fibre density moving from the centre to the periphery of the culm and a corresponding stiffness gradient (low to high stiffness from culm centre to periphery) [55,56]. This gradient gives bamboo its characteristic flexural response, allowing it to resist bending in high winds. In contrast to bamboo, which has homogeneous, single transverse and longitudinal functional gradients, the gradients in *S. lepidophylla* are more complex and show heterogeneity not only between tissue types but also within a given tissue. Thus, it is interesting to observe how rearranging the geometry or changing the level of homogeneity of functional gradients can give rise to distinct mechanical and deformational behaviours. This observation can provide insight into the design of devices with complex shape-shifting and mechanical responses.

The three-dimensional interplay of tissue and cell wall functional gradients in *S. lepidophylla* also highlights appealing features that could be deliberately manipulated to produce synthetic actuators with superior functionality. Careful incorporation of spatial inhomogeneity into active bilayer systems can confer characteristics that can direct fluid-responsive conformational changes. Directional deformation attained through this basic strategy could be integrated with other paradigmatic concepts, such as origami, to potentially generate distinct conformational states depending on the type or level of stimulus (e.g. water) applied to the structure [15,57–59]. Curvature changes, shape-shifting and dimensional transformations can serve multiple sectors, where the requirements of folding, packaging and deployment are paramount, such as in aerospace components (e.g. self-deploying satellites) [60,61], self-folding medical devices and drug delivery systems (e.g. drug release) [62,63] and architectural design of environmentally responsive buildings (e.g. self-opening windows) [9,64]. In terms of implementation, computational models would help decipher the interaction between morphological and compositional gradients, and how these features could be an asset for the design of synthetic systems [65–67]. This would be a starting point for the realization of proof-of-concept prototypes for novel multi-objective, programmable origami composites and metamaterials with uses in a variety of applications [11,68–70].

## Supplementary Material

Supplementary Figures and Tables

## Supplementary Material

Movie S1

## Supplementary Material

Movie S2

## Data accessibility

Data related to this paper are available as electronic supplementary material.
