# Supplementary material for: Three-dimensional functional gradients direct stem curling in the resurrection plant Selaginella lepidophylla
Source: J R Soc Interface. 2019 Oct 30;16(159):20190454. doi: 10.1098/rsif.2019.0454 (PMC6833318; doi:10.1098/rsif.2019.0454)
Supplement: Supplementary Figures and Tables [file rsif20190454supp1.pdf]

Supplementary Information for

Three-dimensional functional gradients direct stem curling in the resurrection plant *Selaginella lepidophylla*

Journal of the Royal Society Interface

V. Brulé, A. Rafsanjani, M. Asgari, T. L. Western and D. Pasini

Corresponding Authors: Tamara L. Western and Damiano Pasini
McGill University, Montréal, QC, Canada
E-mail: tamara.western@mcgill.ca & damiano.pasini@mcgill.ca

This PDF file includes:
Figures S1-S3
Tables S1-S4
Captions for Movies S1-S2
References for SI citations.

Other supplementary materials for this manuscript include the following:
Movies S1-S2.

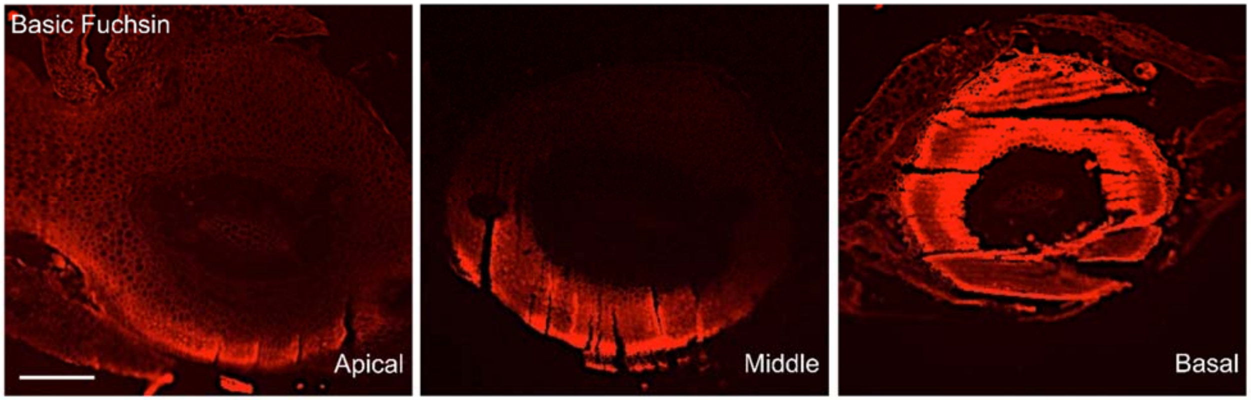

Fig. S1. Tissue Lignification as Detected with Basic Fuchsin. Apical cross-sections show a higher degree of tissue lignification in abaxial cortex, near the periphery of the stem. Middle cross-sections are lignified throughout the abaxial cortex. Uniform lignification is observed in both adaxial and abaxial cortex in basal cross-sections. Scale bar: 200 µm.

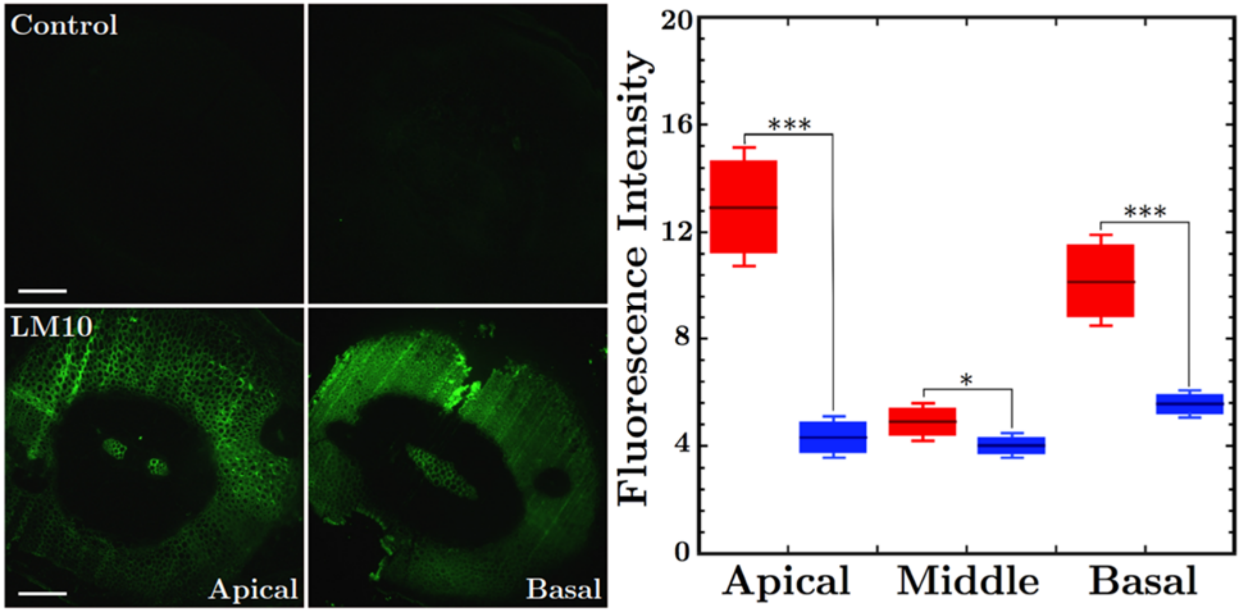

Fig. S2. LM10 Binding Pattern. Control (first row) cross-sections incubated with secondary antibody show little to no fluorescence signal. LM10 (second row) binds in a pattern similar to LM11 (Figure 3e), and shows similar fluorescence intensity (boxplot) patterns between adaxial and abaxial stem sides, and from stem tip-to-base. Scale bars: 200 µm.

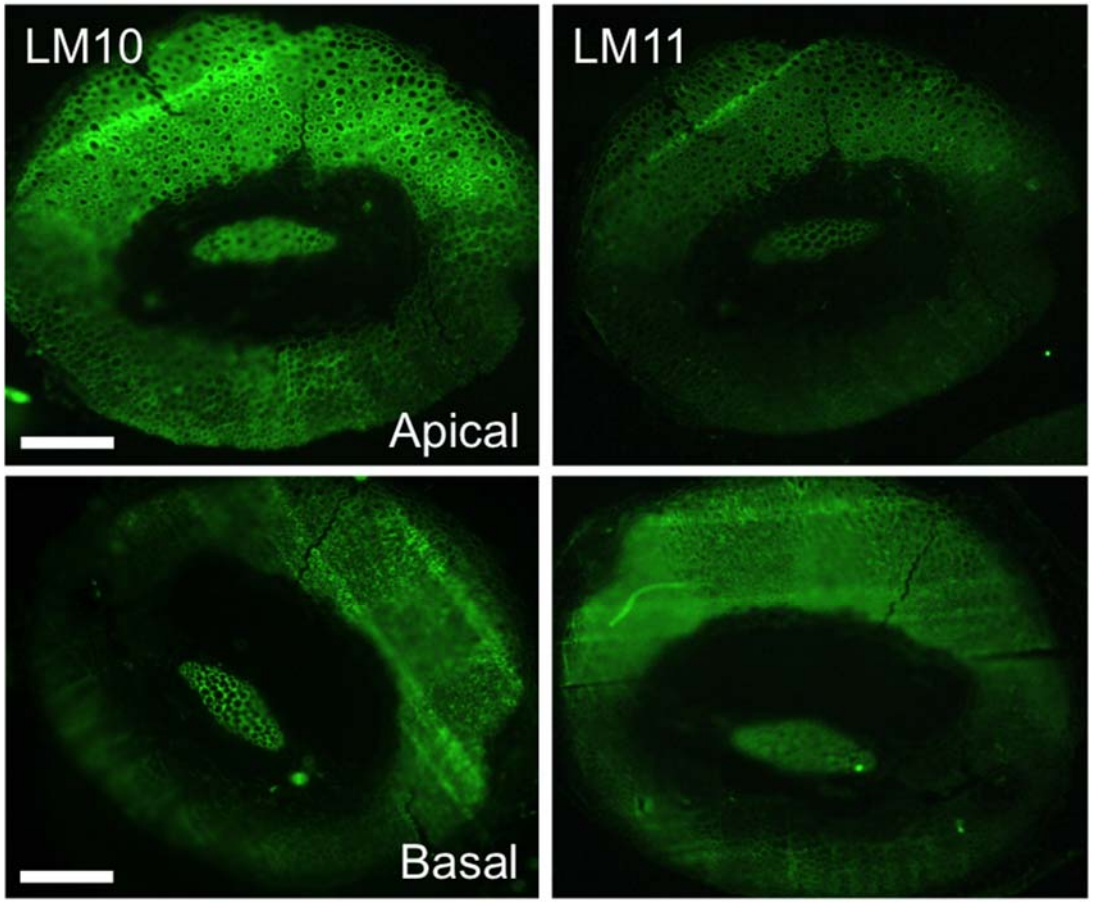

Fig. S3. Antibody Binding Pattern in Outer Stem Cross-Sections. Outer stems show similar binding patterns between LM10 and LM11 antibodies, as well as in tip-to-base cross-sections (middle section not shown). Scale bar: 200 µm.

TABLE S1. *S. lepidophylla* Stem Dimensions (Mean ± Standard Error).

	Width	Thickness	Length
Adaxial	1.93 ± 0.08	**0.77 ± 0.06**[+]	4.65 ± 0.22
Abaxial	1.78 ± 0.11	**0.41 ± 0.06**[+]	4.65 ± 0.21

Dimensions (width and thickness in mm, length in cm) for separated adaxial and abaxial stem sides were measured and recorded prior to tensile testing. Differences between adaxial and abaxial dimensions were tested using two-sided Wilcoxon sign-rank tests with a cut-off of p=0.05. Fifteen pairs (adaxial and abaxial portions from fifteen individual stems) were tested. Significant results (i.e., p<0.05) are marked by +.

TABLE S2. Mechanical Properties of *S. lepidophylla* Stems in MPa (Mean ± Standard Error).

**Whole Stem**	**Young's Modulus (E)**	**Yield**	**Upper Tensile Strength**	**Failure**	**Modulus of Resilience**
Inner	288.08 ± 28.39	281.45 ± 27.23	258.77 ± 23.49	255.74 ± 23.61	144.33 ± 16.75
Outer with Microphylls	483.40 ± 55.79	399.83 ± 45.17	396.71 ± 41.55	377.07 ± 40.39	173.94 ± 20.83
Outer without Microphylls	511.83 ± 49.17	430.72 ± 48.10	403.62 ± 40.51	377.29 ± 40.93	188.07 ± 25.94
Tukey's HSD*					
1 vs 2	**0.01**+	0.11	**0.02**+	0.05	0.60
1 vs 3	**0.00**+	**0.03**+	**0.02**+	0.05	0.33
2 vs 3	0.90	0.86	0.99	1.00	0.89

Tensile tests were performed on inner and outer stem types, and various mechanical properties were calculated from stress-strain graphs. Differences among stem types were tested using one-way ANOVA (analysis of variance) with a cut-off of p=0.05. Twenty-five stems of each stem type were tested. Differences among stem types were further tested with a Tukey’s HSD (honestly significant difference) analysis. Significant results (i.e., p<0.05) are marked by +. *: 1= Inner, 2=Outer with Microphylls, 3=Outer without Microphylls.

TABLE S3: Mechanical Properties of Inner *S. lepidophylla* Stem Tissue in MPa (Mean ± Standard Error).

Tissue Region	Young's Modulus (E)	Yield	Upper Tensile Strength	Failure	Modulus of Resilience
**Adaxial**+	269.74 ± 28.44	194.45 ± 32.58	194.28 ± 32.03	209.72 ± 24.61	90.32 ± 31.73
**Abaxial**+	876.80 ± 113.46	1031.59 ± 190.45	1045.89 ± 190.86	1006.23 ± 185.14	1037.78 ± 448.30
Control*	809.21 ± 223.51	502.51 ± 154.48	455.13 ± 119.70	406.32 ± 123.35	179.02 ± 68.09

Differences between adaxial and abaxial stem regions for each mechanical property were tested using two-sided Wilcoxon sign-rank tests with a cut-off of p=0.05. Fifteen pairs (adaxial and abaxial portions of an individual stem) were tested. Significant differences (marked by +) were found between adaxial and abaxial regions for all properties tested. *: Control tissues (left and right stem sides) were also similarly tested. No significant difference existed between left and right sides; thus, both sides were grouped as a single control.

TABLE S4. *S. lepidophylla* Tissue and Cell Wall Composition.

Antibody[a-c]	Polysaccharide	Epitope	Present	Gradient Apical to Basal	Gradient Adaxial to Abaxial	Location & Tissue Type**
**LM5[a]**	Pectin	Linear (1-4)-β-D-galactosyl residues	-	-	-	-
**LM6[a]**	Pectin	Linear (1-5)-α-L-arabinan	-	-	-	-
**LM7[a]**	Pectin	Partially methyl-esterified homogalacturonan	-	-	-	-
**LM10[b]**	Hemicellulose	Unsubstituted/low substituted xylan	+	+	+	SCW Cortical
**LM11[b]**	Hemicellulose	Unsubstituted/low/highly substituted xylan	+	+	+	SCW Cortical
**LM18[c]**	Pectin	Unesterified/partially methyl-esterified homogalacturonan	-	-	-	-
**LM20[c]**	Pectin	Methyl-esterified homogalacturonan	-	-	-	-
**JIM5[a]**	Pectin	Unesterified/partially methyl-esterified homogalacturonan	+	-	-	ML Metaxylem
**JIM7[a]**	Pectin	Methyl-esterified homogalacturonan	+	-	-	ML Metaxylem | PCW Phloem
**JIM13[a]**	Hemicellulose	Arabinogalactan, AGPs*	+	-	-	PCW Trabeculae
**JIM14[a]**	Hemicellulose	Arabinogalactan, AGPs	-	-	-	-
**M7[a]**	Pectin	Rhamnogalacturonan I	-	-	-	-
**M14[a]**	Pectin	Unbranched rhamnogalacturonan I	-	-	-	-
**M15[a]**	Hemicellulose	Arabinogalactan on sidechains of rhamnogalacturonan I, AGPs	+	-	-	ML Metaxylem | SCW Cortical
**M36[a]**	Pectin	Unbranched rhamnogalacturonan I	-	-	-	-
**M38[a]**	Pectin	Fully de-esterified homogalacturonan	+	-	-	ML Metaxylem | PCW Phloem | SCW Cortical
**M58[a]**	Hemicellulose	Xyloglucan	+	-	-	ML Cortical | PCW Phloem
**M89[a]**	Hemicellulose	Xyloglucan (except XXXG)	+	-	-	ML Cortical | PCW Phloem | SCW Cortical
**M100[a]**	Hemicellulose	Xyloglucan subunit XXXG	+	-	-	ML Cortical
**M118[a]**	Hemicellulose	Monocot xylans	-	-	-	-

*: AGP= arabinogalactan. **ML=middle lamella; PCW=primary cell wall; SCW=secondary cell wall. Twenty antibodies specific to various hemicellulose and pectin epitopes, from three different antibody series (LM, JIM, and M) were tested on cross-sections from apical, middle, and basal regions of inner stems. The presence (+) or absence (-) of a binding signal was recorded, as well as the presence/absence of gradient binding patterns (tip-to-base, and adaxial-to-abaxial). The cell wall location (PCW, SCW, or ML) and tissue type (cortex or vasculature) where the signal was observed was also recorded. a–c: (a) Complex Carbohydrate Research Centre [1], (b) Plant Probes [2], and (c) Plant Probes [3].

Other supplementary materials available for this manuscript on the journal website include the following:

Movie S1.
Time-lapse videos (sped up 2400x) showing inner and outer stem unfolding/folding patterns through two cross-sections of mature *Selaginella lepidophylla* plants rehydrating (cross-section 1) and dehydrating (cross-section 2) over the course of six hours. Older (i.e., outer) stems on the periphery of the plant unfold/fold first, followed by more gradual unfolding/folding of younger (i.e., inner) stem layers. Complete rehydration/dehydration and unfolding/folding of the entire plant requires approximately twenty-fours hours.

Movie S2.
Time-lapse videos (sped up 2400x) showing individual inner and outer stems unfolding/folding over the course of six-hour rehydration and dehydration cycles. Older (i.e., outer) stems curl to an arc shape, whereas inner stems curl into a tight spiral. The deformation patterns remain consistent across repeated wetting and drying of the stem tissue (described in more detail in [4]).

References

1. Pattathil S, et al. (2010) A comprehensive toolkit of plant cell wall glycan-directed monoclonal antibodies. Plant Phys 153(2):514–525.
2. McCartney L, Marcus SE, & Knox JP (2005) Monoclonal antibodies to plant cell wall xylans and arabinoxylans. J Histochem Cytochem 53(4):543–546.
3. Hall HC, Cheung J, Ellis BE (2013) Immunoprofiling reveals unique cell-specific patterns of wall epitopes in the expanding Arabidopsis stem. Plant J 74(1):134–147.
4. Rafsanjani, A., Brulé, V., Western, T. L., & Pasini, D. Hydro-responsive curling of the resurrection plant Selaginella lepidophylla. Sci. Rep. 5, 8064 (2015).
